# Micro-RNA expression in cisplatin resistant germ cell tumor cell lines

**DOI:** 10.1186/1476-4598-10-52

**Published:** 2011-05-15

**Authors:** Matthias Port, Stephanie Glaesener, Christian Ruf, Armin Riecke, Carsten Bokemeyer, Viktor Meineke, Friedemann Honecker, Michael Abend

**Affiliations:** 1 Department of Hematology, Hemostasis, Oncology, and Stem Cell Transplantation, Hannover Medical School, Hannover, Germany; 2 Department of Oncology/Hematology/Bone Marrow Transplantation with the section of Pneumology, University Hospital Hamburg Eppendorf, Germany; 3 Department of Urology, Federal Armed Forces Hospital, Hamburg, Germany; 4 Department of Internal Medicine, Federal Armed Forces Ulm, Ulm, Germany; 5 Bundeswehr Institute of Radiobiology, Munich, Germany

**Keywords:** germ cell tumor cell line, cisplatin resistance, microRNA, gene expression

## Abstract

**Background:**

We compared microRNA expression patterns in three cisplatin resistant sublines derived from paternal cisplatin sensitive germ cell tumor cell lines in order to improve our understanding of the mechanisms of cisplatin resistance.

**Methods:**

Three cisplatin resistant sublines (NTERA-2-R, NCCIT-R, 2102EP-R) showing 2.7-11.3-fold increase in drug resistance after intermittent exposure to increasing doses of cisplatin were compared to their parental counterparts, three well established relatively cisplatin sensitive germ cell tumor cell lines (NTERA-2, NCCIT, 2102EP). Cells were cultured and total RNA was isolated from all 6 cell lines in three independent experiments. RNA was converted into cDNA and quantitative RT-PCR was run using 384 well low density arrays covering almost all (738) known microRNA species of human origin.

**Results:**

Altogether 72 of 738 (9.8%) microRNAs appeared differentially expressed between sensitive and resistant cell line pairs (NTERA-2R/NTERA-2 = 43, NCCIT-R/NCCIT = 53, 2102EP-R/2102EP = 15) of which 46.7-95.3% were up-regulated (NTERA-2R/NTERA-2 = 95.3%, NCCIT-R/NCCIT = 62.3%, 2102EP-R/2102EP = 46.7%). The number of genes showing differential expression in more than one of the cell line pairs was 34 between NTERA-2R/NTERA-2 (79%) and NCCIT-R/NCCIT (64%), and 3 and 4, respectively, between these two cell lines and 2102EP-R/2102EP (about 27%). Only the has-miR-10b involved in breast cancer invasion and metastasis and has-miR-512-3p appeared to be up-regulated (2-3-fold) in all three cell lines. The hsa-miR-371-373 cluster (counteracting cellular senescence and linked with differentiation potency), as well as hsa-miR-520c/-520h (inhibiting the tumor suppressor p21) were 3.9-16.3 fold up-regulated in two of the three cisplatin resistant cell lines. Several new micro-RNA species missing an annotation towards cisplatin resistance could be identified. These were hsa-miR-512-3p/-515/-517/-518/-525 (up to 8.1-fold up-regulated) and hsa-miR-99a/-100/-145 (up to 10-fold down-regulated).

**Conclusion:**

Examining almost all known human micro-RNA species confirmed the miR-371-373 cluster as a promising target for explaining cisplatin resistance, potentially by counteracting wild-type P53 induced senescence or linking it with the potency to differentiate. Moreover, we describe for the first time an association of the up-regulation of micro-RNA species such as hsa-miR-512-3p/-515/-517/-518/-525 and down-regulation of hsa-miR-99a/-100/-145 with a cisplatin resistant phenotype in human germ cell tumors. Further functional analyses are warranted to gain insight into their role in drug resistance.

## Background

Cisplatin (cis-diamminedichloroplatinum II, CDDP) is a chemotherapeutic agent widely used in the treatment of several solid tumors, among them testicular cancer, lung cancer, breast cancer, and bladder cancer. Resistance to cisplatin is a serious obstacle to effective cancer therapy. Clinically relevant levels of resistance can emerge quickly after treatment. Beside intrinsic resistance, acquired or gradually developing resistance has been observed in tumors under therapy. Several mechanisms underlying resistance have been described, like decreased exposure to the drug, e.g. via reduced drug accumulation/drug-target interaction or increased detoxification response, diminished cell-cycle effects, reduced apoptotic responses, or increased DNA-repair [1,2]. A number of these biological processes are controlled on a post-transcriptional level by noncoding micro-RNA species. We created an *in vitro *model of acquired cisplatin resistance by long term exposure of three well established germ cell tumor cell lines to cisplatin, resulting in sublines with significantly increased resistance to cisplatin. The paternal cell lines are (1) the p53-wt (wild type) pluripotent gonadal germ cell tumor cell line NTERA-2, (2) the p53-mutated pluripotent extragonadal nonseminomatous germ cell tumor cell line NCCIT and (3) the p53-wt nullipotent embryonal cell carcinoma 2102EP, derived from a primary human testicular teratocarcinoma. NCCIT cells have previously been shown to have an approximately 4-fold higher IC_50 _to cisplatin, compared to NTERA-2 [3]. We utilized a quantitative RT-PCR (RTQ-PCR) based platform for detection of almost all currently known (738) human micro-RNA species on these 6 cell lines. Our approach was designed to characterize the role of micro-RNAs on the presumably multifactorial phenomenon of acquired cisplatin resistance in germ cell tumors.

## Methods

### 1 Cell lines

Both chemo sensitive paternal 2102EP (PW Andrews, Sheffield, U.K.) and NCCIT (ATCC, Manassas, VA, USA) cells as well as their cisplatin resistant sublines were cultured in DMEM F12 (1:1) medium containing 10% fetal calf serum (Gibco-BRL, Invitrogen, U.K.). NTERA-2 (DSMZ, Braunschweig, Germany) and the cisplatin resistant subline were cultured in DMEM medium supplemented with Glutamax-I (Gibco-BRL, Invitrogen, U.K.) containing 10% fetal calf serum (Gibco-BRL, Invitrogen, U.K.). Cells were incubated at 37°C in a humidified atmosphere with 5% CO_2 _and were passaged every 3-4 days. After trypsination of cultured cells from steady state conditions and at comparable cell density, aliquots containing 10 × 10^6 ^cells were transferred into 1 ml RNA-later solution and stored at -20°C. Three aliquots per cell line originating from different cell passages (n+2, n+4 and n+5) were processed and stored as described above.

### 2 Induction of cisplatin resistance

The cisplatin resistant sublines NTERA-2-R, 2102EP-R and NCCIT-R were generated by intermittent exposure of the parental cell lines to cisplatin over a time period of 18 month, starting with the respective IC_10 _dose of each parental cell line. At reaching 50% cell kill, the addition of cisplatin was interrupted and cells were allowed to recover over three passages. Analyses presented here were performed after maintaining the resistant sublines for at least 1 week in cisplatin-free medium to allow for an adequate wash-out period. Results of cytotoxicity experiments of all six cell lines using the MTT assay (3-(4,5-Dimethylthiazol-2-Yl)-2,5-Diphenyltetrazolium Bromide) have been published [4] The experiments were performed in 3 completely independent replicates for every cell line including generation of resistant cell lines, cell culture, RNA isolation and RTQ-PCR measurements.

### 3 RNA Isolation

Cells were transported on dry ice in RNA-later solution (Qiagen, Hilden, Germany) and stored at -80°C until use. After thawing the cells, they were disrupted using a denaturing lysis buffer, and RNA was isolated by combining organic extraction (phenol-chloroform) followed by immobilization of RNA on glass-fiber filters employing the mirVana miRNA Isolation Kit (Ambion, Applied Biosystem, Weiterstadt, Germany) in order to purify total RNA including small RNA species. Remaining DNA was digested on glass-fiber filters (RNase free DNase Set,Qiagen, Hilden, Germany), and after performing quality controls, RNA was stored at -80°C until analysis by RTQ-PCR.

To control the quality and purity (protein or DNA contamination) of isolated total RNA including small RNA, we performed spectral photometry, agarose gel electrophoresis, and PCR (using β-Actin primers). Only high quality total RNA including small RNA species was used for further experiments.

### 4 RTQ-PCR with "Low Density Array" (LDA)

Aliquots from each RNA sample (10 × 1 μg) were reversely transcribed without preamplification over three hours using the so called *"Megaplex pools without preamplification protocol for microRNA expression analysis protocol"*. In a second step, the whole template cDNA and 450 μl 2x RT-PCR master mix were adjusted to a total volume of 900 μl by adding nuclease free water, and aliquots of 100 μl were pipetted into each fill port of a 384-well LDA. Cards were centrifuged twice (12,000 rpm, 1 min, Multifuge3S-R, Heraeus, Germany), sealed, transferred into the 7900 RTQ-PCR instrument and a specific RTQ-PCR protocol was run over two hours using the 384-well LDA format. Two different LDAs existed, thus covering 738 human micro-RNAs. This made it necessary to create two kinds of cDNAs suitable for each of both LDAs using different sets of primers.

Normalization was performed using the median gene expression on each LDA separately, because this proved to be the more robust and slightly more precise method compared to a normalization approach using a housekeeping micro-RNA species provided on the LDA (data not shown). The median gene expression was subtracted from the CT-value of each of the spotted genes, following the ∆CT-quantitative approach for normalization purposes. Normalized gene expression results of cisplatin-resistant cell lines were expressed relative to the genes measured in the paternal chemosensitive cell lines by subtracting the corresponding CT-values (∆∆CT-quantitative approach). As a result, differential gene expression of cisplatin-resistant cell lines was expressed as a several-fold up- or down regulation of micro-RNAs relative to their paternal chemo sensitive origin. These ratios of corresponding cell line pairs are expressed such as e.g. NTERA-2-R/NTERA-2. Only ratios ≥2/≤0.5 were considered to represent differentially expressed genes. All the materials and instruments used for RTQ-PCR were ordered from Applied Biosystems, Weiterstadt, Germany.

All technical procedures were performed in accordance to standard operating procedures (SOP) implemented in our laboratory in 2008 when the Institute became accredited according to DIN EN ISO 9001/2008.

### 5 Statistics

Statistical measures (e.g. coefficient of variation, CV and 25-75% quantiles) were computed using SAS (release 9.1.3, Cary NC, USA).

## Results

Intermittently culturing NTERA-2, 2102EP, and NCCIT cell lines exposed to increasing concentrations of cisplatin resulted in a 2.7 - 11.3 increase in IC_50 _to the substance, as measured by the MTT assay (3-(4,5-Dimethylthiazol-2-Yl)-2,5-Diphenyltetrazolium Bromide). Whereas the IC_50 _values of the sensitive parental cell lines were 0.45 μM (+/-0.1 standard deviation, SD), 0.75 μM (+/- 0.1), and 1.75 μM (+/- 0.1), respectively, the three cisplatin resistant sublines exhibited IC_50 _values of 5.1 μM (+/- 0.1) in NTERA-2R, 4.9 μM (+/- 0.1) in 2102EP-R, and 4.7 μM (+/- 0.2 ) in NCCIT-R. Results of the cytotoxicity experiments have been reported elsewhere [4].

The coefficient of variation (CV, measure of standard deviation variability expressed in percent relative to the mean differential gene expression for each micro-RNA) was chosen to describe the observed methodological, intra- and interindividual variability of our three independently performed experiments. Mean CV values of differentially expressed genes and associated interquartile ranges appeared cell line dependent, showing increased mean CV values (25-75 percentile) starting at 25.3% (25-75 percentile: 15.7-29.0) in cell line pairs such as NCCIT-R/NCCIT, 30.5% (25-75 percentile: 18.5-36.3) in NTERA-2-R/NTERA-2, and 37.4% (25-75 percentile: 18.6-40.4) in 2102EP-R/2102EP cells (table 1). On average, mean CV did not exceed 30% (28.9%).

**Table 1:** Differential gene expression relative to the corresponding gene of the paternal cell line

	Cell lines			
CV distribution	*NTERA-2-R/NTERA-2*	*NCCIT-R/NCCIT*	*2102EP-R/2102EP*	*together*
mean	30.5	25.3	37.4	28.9
median	27.3	21.7	32.7	24.1
25-75 percentile	18.5-36.3	15.7-29.0	18.6-40.4	16.5-33.8
n	43	53	15	111

The coefficient of variation (CV, chosen as a measure of standard deviation variability expressed in percent relative to the mean differential gene expression) was calculated for each micro-RNA species. Parameters of CV distribution such as mean, median and first-third quartile range were calculated and depicted for each cell line pair consisting of the cisplatin-resistant (-R added to the paternal cell line's name) relative to the cisplatin-sensitive paternal cell line as well as summarized.

**Figure 1:**
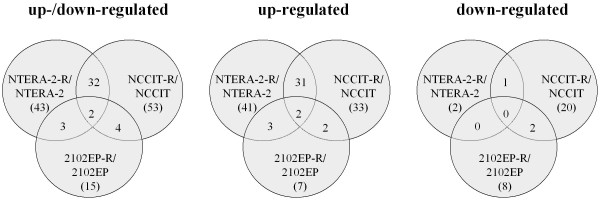
**Venn diagram on total number (in parenthesis) and overlapping number of differentially expressed genes calculated in cell line pairs consisting of the cisplatin-resistant (-R added to the paternal cell line's name) relative to the cisplatin-sensitive paternal cell line**.

About 30-45% of the 738 microRNAs examined showed C_T_-values within the linear dynamic range of the method (CT 7-29, data not shown). Altogether 72 of 738 genes appeared differentially expressed (table 2) with 43, 53 and 15 genes found in NTERA-2-R/NTERA-2, NCCIT-R/NCCIT and 2102EP-R/2102EP cell line pairs, respectively (figure 1). On average 72% of the genes were up-regulated in resistant compared to sensitive cells, a feature that was more prominent in NTERA-2-R/NTERA-2 (41 of 43 genes, 95.3%) compared to NCCIT-R/NCCIT (33 of 53 genes, 62.3%) or even 2102EP-R/2102EP (7 of 15 genes, 46.7%) cell pairs (table 2). The number of down-regulated genes ranged from 4.7% in NTERA-2-R/NTERA-2 (2 of 43 genes) to 37.7% in NCCIT-R/NCCIT (20 of 53 genes) and 53.3% in 2102EP-R/2102EP cell line pairs (8 of 15 genes). Thirty-four overlapping genes were found in the two pairs NTERA-2-R/NTERA-2 (34 of 43 genes, 79%) and NCCIT-R/NCCIT (34 of 53 genes, 64%). The overlapping number of differentially expressed genes was only 5 between cell line pairs NTERA-2-R/NTERA-2 and 2102EP-R/2102EP, and 6 between NCCIT-R/NCCIT and 2102EP-R/2102EP (about 27%). Only hsa-miR-10b and has-miR-512-3p species were found to be up-regulated (2-8-fold) in all three cell lines.

**Table 2:** Differential gene expression relative to the corresponding gene of the paternal cell line.

	Mean differential gene expression in cell line pairs										Mean differential gene expression in cell line pairs								
	NTERA-2-R/NTERA-2			NCCIT-R/NCCIT			2102EP-R/2102EP				NTERA-2-R/NTERA-2			NCCIT-R/NCCIT			2102EP-R/2102EP		
Detector	fold-change	SEM	p-value	fold-change	SEM	p-value	fold-change	SEM	p-value	Detector	fold-change	SEM	p-value	fold-change	SEM	p-value	fold-change	SEM	p-value
hsa-miR-708	0.5	0.1	0.014	0.4	0.1	0.004				hsa-miR-525-3p	6.8	1.3	0.009	8.0	0.9	<0.001			
hsa-miR-130b	0.5	0.1	0.096							hsa-miR-519a	6.8	1.0	0.008	6.0	0.9	0.081*			
hsa-miR-455-5p	2.0	0.4	0.059							hsa-miR-518a-3p	6.9	1.1	0.004	7.2	0.3	<0.001			
hsa-miR-193b	2.1	0.5	0.036							hsa-miR-520b	7.0	2.3	0.004	5.5	0.8	<0.001			
hsa-miR-146a	2.1	0.3	0.1*	2.6	0.1	<0.001				hsa-miR-517b	7.2	1.2	0.003	6.4	1.5	0.006			
RNU43	2.2	0.3	0.1*							hsa-miR-518b	7.3	0.4	<0.001	8.1	1.2	<0.001			
hsa-miR-526b	2.2	0.4	0.061							hsa-miR-515-5p	7.8	1.0	0.005	5.6	0.3	<0.001			
hsa-miR-302b	2.3	1.2	0.512	0.5	0.1	0.058				hsa-miR-145				0.0	0.0	<0.001			
hsa-miR-524-3p	2.4	0.1	0.019							hsa-miR-100				0.1	0.0	<0.001			
hsa-miR-10b	2.8	0.6	0.006	2.0	0.6	0.081*	2.7	0.5	0.126	hsa-miR-99a				0.1	0.0	<0.001			
hsa-miR-205	2.9	0.5	0.004	0.5	0.1	0.034				hsa-miR-582-5p				0.2	0.0	<0.001			
hsa-miR-518c	2.9	0.4	0.004							hsa-miR-125b				0.2	0.0	<0.001	0.4	0.0	0.012
hsa-miR-801	3.0	1.0	0.172							hsa-miR-450b-5p				0.4	0.0	0.038			
hsa-miR-523	3.5	0.1	0.004	3.0	0.5	0.012				hsa-miR-302d				0.4	0.0	<0.001			
hsa-miR-362-3p	3.7	1.7	0.026				2.4	0.8	0.132	hsa-miR-192				0.4	0.1	0.004			
hsa-miR-520f	3.7	0.3	0.010	2.4	0.3	0.001				hsa-miR-218				0.5	0.1	0.030	0.3	0.0	<0.001
hsa-miR-520h	3.9	0.4	<0.001	7.1	0.7	<0.001				hsa-miR-335				0.5	0.1	0.062			
hsa-miR-371-3p	4.0	0.3	<0.001	15.2	2.1	<0.001				hsa-miR-21				0.5	0.0	0.061			
hsa-miR-373	4.1	0.7	0.006	12.0	2.9	<0.001				hsa-miR-576-3p				0.5	0.1	0.001			
hsa-miR-519c-3p	4.3	0.3	0.008	3.2	0.3	0.001				hsa-miR-363				0.5	0.0	0.001			
hsa-miR-522	4.3	0.7	0.002	6.4	0.4	0.002				hsa-miR-424				0.5	0.0	0.002			
hsa-miR-526b	4.3	0.5	<0.001	8.3	2.3	0.002				hsa-miR-302a				0.5	0.0	0.003			
hsa-miR-519b-3p	4.4	0.4	0.006	7.8	1.0	0.003				hsa-miR-194				0.5	0.2	0.078			
hsa-miR-518d-5p	4.5	0.5	0.004	3.3	0.4	0.001				hsa-miR-221				0.5	0.1	0.034			
hsa-miR-516b	4.5	0.4	0.002	3.1	0.5	0.002				hsa-miR-935				2.5	0.1	<0.001			
hsa-miR-512-5p	4.6	1.3	0.021	6.3	0.8	0.001				hsa-miR-383				13.3	2.8	<0.001			
hsa-miR-520c-3p	4.6	0.6	<0.001	5.6	0.5	0.002				hsa-miR-489							0.1	0.0	<0.001
hsa-miR-372	4.7	1.0	0.081*	16.3	2.0	<0.001				hsa-miR-365							0.3	0.1	<0.001
hsa-miR-512-3p	5.1	0.9	0.004	7.8	1.2	<0.001	2.1	0.2	0.024	hsa-miR-193a-5p							0.4	0.1	0.008
hsa-miR-518f	5.2	0.7	0.003	6.3	0.9	<0.001				hsa-miR-296-5p							0.4	0.0	0.003
hsa-miR-519d	5.2	0.7	0.001	7.0	2.6	0.003				hsa-miR-484							0.4	0.0	0.002
hsa-miR-517a	5.3	1.2	0.005	6.0	0.6	<0.001				hsa-miR-335*							0.5	0.0	0.007
hsa-miR-520g	5.8	0.6	0.005	7.5	1.5	<0.001				hsa-miR-34a							2.4	0.4	0.048
hsa-miR-517c	6.1	0.5	0.001	6.7	0.5	<0.001				hsa-miR-923							2.4	0.6	0.188*
hsa-miR-515-3p	6.4	0.2	<0.001	4.8	0.5	<0.001				hsa-miR-146b-5p							3.9	0.6	0.081
hsa-miR-518e	6.7	0.4	0.001	8.1	1.5	<0.001				hsa-miR-19b-1							5.7	3.5	0.081

Differential gene expression (mean fold-change from three independent experiments) was calculated from gene expression measured in the chemoresistant cell line (-R added to the paternal cell line's name) relative to the corresponding gene of the paternal cell line. Genes are listed in ascending order of the NTERA-2 cell's fold-change followed by NCCIT and 2102EP values. P-values were calculated with t-test (equal variance and normal distributed data) and values with asterix with the MannWhitney rank sum test.

Taken together, NTERA-2-R/NTERA-2 and NCCIT-R/NCCIT shared the vast majority of their differentially altered micro-RNAs, whereas 2102EP-R/2102EP showed a rather different profile. A complete list of all detectable miRNA examined is provided as additional file (additional file 1: Table S1).

## Discussion

Our approach enabled us to investigate almost all currently known human micro-RNAs simultaneously. Quantitative RT-PCR was chosen because of it's recognized precision and superiority over other methods for gene expression analysis [5-7]. Our experiments were run in triplicate and on average revealed a CV <30% covering variability caused by methodological (mean CV <5%, data not shown), intra- and inter-individual sources (table 1). Since this CV is clearly below the definition of a two-fold threshold for differential gene expression, we are convinced that the results of our analysis represent true differences in micro-RNA expression between the cell line pairs.

Of 738 micro-RNA species examined, only 9.8% (72) appeared differentially expressed with considerable overlap in micro-RNA gene expression occurring between the cell line pairs NTERA-2-R/NTERA-2 and NCCIT-R/NCCIT, but considerably less overlap with the cell line pair 2102EP-R/2102EP cells (table 1).

Functionally, the most striking distinguishing feature between the cell lines NTERA-2 and NCCIT on the one hand, and 2102EP on the other hand is a difference in their capacity to differentiate. Whereas the former two lines can differentiate upon stimuli such as exposure to retinoid acid, the latter is nullipotent and is therefore devoid of alterations induced by processes of cellular differentiation [8]. Interestingly, there is increasing evidence for a link between cellular differentiation and resistance to chemotherapy in germ cell tumors, both in cell line models, as well as in primary tumor material, which seems to be linked to two microRNA clusters, namely miR371-373 and miR302 [9].

In contrast to many features shared by NTERA-2 and NCCIT, extragonadal NCCIT cells show a higher resistance level to cisplatin, compared to NTERA-2 [3]. In this context, the several fold-change differences measured in the micro-RNA cluster covering hsa-miR-371-373 is of interest. Based on our findings, hsa-miR-371-373 expression in NTERA-2-R increased 4.0-4.7-fold relative to the paternal cisplatin sensitive NTERA-2 line, and even more in NCCIT-R, relative to the cisplatin sensitive paternal NCCIT cells, showing an 12.0-16.3-fold upregulation, table 1. Noteworthy, this micro-RNA cluster has been discussed in the context of the presence of wild-type p53 in germ cell tumors, counteracting tumorigenesis by induction of senescence [2,10,11]. By up-regulation, the cluster prevents p53-driven cellular senescence via a plethora of target genes, e.g. NEO1 or LATS2 [2], therefore leading to cell proliferation even in the presence of wild-type p53. Furthermore, this cluster has been described in cells exhibiting stem cell properties [12]. Noteworthy, this cluster has recently been identified by other authors to be the most significant differentially expressed microRNA in human germ cell tumors [13]. Therefore, it appears a promising target for further analysis in germ cell tumors, e.g. by posttranscriptional silencing with siRNA, to examine the functional role of the cluster in altering gene expression and thereby elucidate it's contribution to the development of cisplatin resistance.

As already mentioned, there are other gene changes which have been discussed in the context of cisplatin resistance [2]. The tumor suppressor p21, regulating transition through the cell cycle and acting downstream of p53, has already been associated with hsa-miR520 belonging to the miR-106/302 family [14]. An increased expression of miR-106/302 family members inhibits the tumor suppressor p21 and rescues human mammary epithelial cells from Ras-induced senescence [14]. In our analysis, hsa-miR520c as well as hsa-miR520h were up-regulated (3.9-7.1-fold) in NTERA-2-R/NTERA-2 and NCCIT-R/NCCIT cell line pairs, which could point towards another mechanism counteracting senescence.

In an *in vitro *model of cisplatin resistance of a squamous cell carcinoma cell line, has-miR-21 was found to be down-regulated in cisplatin resistant cells [15]. In our cell line pairs, significant down-regulation of has-miR-21 was observed in NCCIT-R compared to sensitive NCCIT cells. Furthermore, hsa-miR-146a, a microRNA targeting BRCA1, was the most highly up-regulated microRNA in another *in vitro *model of cisplatin resistance of the breast cancer cell line MCF7 [16]. Similarly, both NTERA-2-R/NTERA-2 and NCCIT-R/NCCIT showed up-regulation in our analysis, yet to a lesser extent. However, has-miR-221, which was also highly up-regulated in cisplatin resistant MCF7 cells, was down-regulated in NCCIT-R and unaltered in the other two cell lines in our experiments, indicating that cisplatin resistance seems to be cell line or tumor type specific. In a lymphoblastic leucemia cell line overexpression of hsa-miR-125b conferred a survival advantage through inhibition of caspase 3 activation after exposure to a broad spectrum of apoptotic stimuli [17]. In our analysis, however, hsa-miR-125b appeared to be up to 5-fold down-regulated in NCCIT-R/NCCIT and 2102EP-R/2102EP cell line pairs, thus making an inhibition of apoptosis through this micro-RNA species unlikely.

According to our analysis, further micro-RNA species appeared either about 8-fold up-regulated (hsa-miR-512-3p/-515/-517/-518/-525) or about 10-fold down-regulated (hsa-miR-99a/-100/-145) in both NTERA-2-R/NTERA-2 and NCCIT-R/NCCIT cell line pairs. Literature results describing a role of these micro-RNAs in cisplatin resistance are missing, warranting further studies to elucidate their potential role in development of resistance.

Recently, a role of the miR-106b seed family members in cisplatin resistance of testicular cancer has been described by another group [18]. Although detectable, neither miRNA-106b, nor miRNA-106a, miRNA-17-5, miRNA-93, and miRNA-20 were differentially expressed in our model. In our view, this points at the multifactorial nature of cisplatin resistance in germ cell tumors.

## Conclusions

In summary, our approach, simultaneously examining almost all known human micro-RNA species, confirms that the miR-371-373 cluster seems to be involved in cisplatin resistance in germ cell tumors in vitro and could be an interesting target for interference with drug resistance. Moreover, new micro-RNA species such as hsa-miR-512-3p/-515/-517/-518/-525 or hsa-miR-99a/-100/-145, also potentially involved in cisplatin resistance, could be identified in the germ cell tumor cell lines studied here. It will be of interest to examine tumor samples of patients with both cisplatin-sensitive and cisplatin-resistant germ cell tumors to analyse whether the changes described here are also found in vivo. Furthermore, functional analyses, e.g. by employing si-RNA and simultaneous whole genome microarrays, can help to examine the causal link with cisplatin resistance and to get insight into the mRNA species controlled by the micro-RNAs.

## Competing interests

The authors declare that they have no competing interests.

## Authors' contributions

MP designed the study, analyzed results and drafted the manuscript. SG carried out experiments related to induction of cisplatin resistance and drafted the manuscript. CR carried out RTQ-PCR experiments and performed statistical analysis. AR carried out RTQ-PCR experiments and analyzed the results. CB participated in the design of the study and coordination. VM participated in the design of the study and coordination. FH was responsible for the induction of cisplatin resistance experiments, designed the study and drafted the manuscript. MA designed the study, was responsible for RTQ-PCR experiments, analyzed results, performed statistical analysis and drafted the manuscript. All authors read and approved the final manuscript.

## Supplementary Material

Additional file 1 **Table S1: Complete list of all detectable miRNA examined**. Differential gene expression relative to the corresponding gene of the paternal cell line - complete list of all detectable miRNAs examined (lying within the linear-dynamic range of our method) Click here for file
