# Supplementary material for: Micro-RNA expression in cisplatin resistant germ cell tumor cell lines
Source: Mol Cancer. 2011 May 15;10:52. doi: 10.1186/1476-4598-10-52 (PMC3120796; doi:10.1186/1476-4598-10-52)
Supplement: Additional file 1 — Table S1: Complete list of all detectable miRNA examined. Differential gene expression relative to the corresponding gene of the paternal cell line - complete list of all detectable miRNAs examined (lying within the linear-dynamic range of our method) [file 1476-4598-10-52-S1.DOC]

**Table S1:** Differential gene expression relative to the corresponding gene of the paternal cell line - complete list of all detectable miRNAs examined (lying within the linear-dynamic range of our method)

| **Mean differential gene expression in cell line pairs** | | | | | | | | |
| --- | --- | --- | --- | --- | --- | --- | --- | --- |
| **NTERA-2-R/ NTERA-2** | | | **NCCIT-R / NCCIT** | | | **2102EP-R / 2102EP** | | |
| Detector | fold-change | SEM | Detector | fold-change | SEM | Detector | fold-change | SEM |
| hsa-let-7e | 1.71 | 0.09 | hsa-let-7e | 0.92 | 0.31 | hsa-miR-100 | 0.71 | 0.20 |
| hsa-miR-100 | 0.94 | 0.23 | hsa-let-7g | 1.03 | 0.20 | hsa-miR-101 | 1.06 | 0.07 |
| hsa-miR-101 | 1.64 | 0.47 | hsa-miR-100 | 0.10 | 0.01 | hsa-miR-103 | 1.08 | 0.16 |
| hsa-miR-103 | 1.13 | 0.05 | hsa-miR-101 | 1.08 | 0.18 | hsa-miR-106a | 1.24 | 0.18 |
| hsa-miR-106a | 1.14 | 0.13 | hsa-miR-103 | 0.65 | 0.08 | hsa-miR-106b | 0.83 | 0.12 |
| hsa-miR-106b | 1.28 | 0.28 | hsa-miR-106a | 0.61 | 0.06 | hsa-miR-106b* | 0.66 | 0.02 |
| hsa-miR-106b* | 0.95 | 0.01 | hsa-miR-106b | 0.72 | 0.07 | hsa-miR-10b | 2.66 | 0.50 |
| hsa-miR-107 | 1.31 | 0.20 | hsa-miR-106b* | 0.81 | 0.06 | hsa-miR-124 | 0.96 | 0.10 |
| hsa-miR-10b | 2.78 | 0.62 | hsa-miR-107 | 0.71 | 0.04 | hsa-miR-125a-3p | 1.15 | 0.10 |
| hsa-miR-124 | 0.78 | 0.11 | hsa-miR-10b | 2.01 | 0.65 | hsa-miR-125a-5p | 0.98 | 0.05 |
| hsa-miR-125a-5p | 1.25 | 0.38 | hsa-miR-125a-5p | 0.97 | 0.03 | hsa-miR-125b | 0.39 | 0.03 |
| hsa-miR-125b | 1.48 | 0.22 | hsa-miR-125b | 0.21 | 0.02 | hsa-miR-126 | 1.19 | 0.13 |
| hsa-miR-126 | 1.08 | 0.03 | hsa-miR-126 | 0.83 | 0.07 | hsa-miR-126* | 0.83 | 0.03 |
| hsa-miR-126* | 1.05 | 0.14 | hsa-miR-126* | 0.80 | 0.09 | hsa-miR-127-3p | 0.96 | 0.12 |
| hsa-miR-128 | 0.88 | 0.18 | hsa-miR-128 | 0.67 | 0.08 | hsa-miR-128 | 0.91 | 0.15 |
| hsa-miR-129-3p | 0.74 | 0.20 | hsa-miR-129-3p | 1.11 | 0.14 | hsa-miR-129-3p | 1.22 | 0.12 |
| hsa-miR-130a | 1.24 | 0.35 | hsa-miR-130a | 0.92 | 0.28 | hsa-miR-130a | 0.91 | 0.10 |
| hsa-miR-130b | 0.92 | 0.06 | hsa-miR-130b | 0.62 | 0.13 | hsa-miR-130b | 1.20 | 0.17 |
| hsa-miR-130b* | 0.48 | 0.07 | hsa-miR-130b* | 0.78 | 0.04 | hsa-miR-130b* | 0.70 | 0.20 |
| hsa-miR-132 | 0.99 | 0.26 | hsa-miR-132 | 1.48 | 0.24 | hsa-miR-132 | 0.91 | 0.11 |
| hsa-miR-135a | 0.96 | 0.19 | hsa-miR-135a | 1.50 | 0.19 | hsa-miR-133a | 0.51 | 0.06 |
| hsa-miR-135a* | 0.72 | 0.05 | hsa-miR-135a* | 1.04 | 0.13 | hsa-miR-134 | 1.21 | 0.14 |
| hsa-miR-135b | 1.19 | 0.14 | hsa-miR-135b | 0.92 | 0.02 | hsa-miR-135a | 0.67 | 0.08 |
| hsa-miR-135b* | 0.79 | 0.05 | hsa-miR-135b* | 0.76 | 0.03 | hsa-miR-135a* | 1.12 | 0.11 |
| hsa-miR-138 | 1.51 | 0.26 | hsa-miR-138 | 1.11 | 0.16 | hsa-miR-135b | 0.96 | 0.07 |
| hsa-miR-138-1* | 1.22 | 0.12 | hsa-miR-138-1* | 1.20 | 0.17 | hsa-miR-135b* | 0.75 | 0.05 |
| hsa-miR-139-5p | 0.59 | 0.11 | hsa-miR-140-3p | 0.91 | 0.09 | hsa-miR-136* | 0.88 | 0.11 |
| hsa-miR-140-3p | 1.03 | 0.17 | hsa-miR-140-5p | 0.81 | 0.10 | hsa-miR-138 | 1.40 | 0.25 |
| hsa-miR-140-5p | 0.92 | 0.11 | hsa-miR-145 | 0.04 | 0.00 | hsa-miR-138-1* | 1.11 | 0.07 |
| hsa-miR-145 | 1.00 | 0.15 | hsa-miR-146a | 2.62 | 0.13 | hsa-miR-139-5p | 0.89 | 0.04 |
| hsa-miR-146a | 2.10 | 0.31 | hsa-miR-146b-5p | 1.43 | 0.20 | hsa-miR-140-3p | 0.97 | 0.15 |
| hsa-miR-146b-5p | 1.66 | 0.20 | hsa-miR-148a | 0.69 | 0.16 | hsa-miR-140-5p | 1.21 | 0.05 |
| hsa-miR-148a | 1.04 | 0.14 | hsa-miR-148b | 0.88 | 0.11 | hsa-miR-141 | 0.58 | 0.03 |
| hsa-miR-148b | 1.20 | 0.33 | hsa-miR-148b* | 0.94 | 0.13 | hsa-miR-141* | 0.97 | 0.17 |
| hsa-miR-148b* | 1.46 | 0.16 | hsa-miR-149 | 1.00 | 0.10 | hsa-miR-145 | 0.75 | 0.10 |
| hsa-miR-149 | 0.82 | 0.09 | hsa-miR-149* | 0.92 | 0.14 | hsa-miR-146a | 0.89 | 0.13 |
| hsa-miR-149* | 0.95 | 0.24 | hsa-miR-150 | 0.84 | 0.20 | hsa-miR-146b-5p | 3.88 | 0.61 |
| hsa-miR-150 | 1.03 | 0.24 | hsa-miR-151-3p | 0.77 | 0.03 | hsa-miR-148a | 1.14 | 0.16 |
| hsa-miR-151-3p | 0.79 | 0.05 | hsa-miR-152 | 0.94 | 0.18 | hsa-miR-148a* | 1.04 | 0.10 |
| hsa-miR-152 | 1.66 | 0.36 | hsa-miR-15a | 0.77 | 0.14 | hsa-miR-148b | 1.03 | 0.09 |
| hsa-miR-15a | 1.23 | 0.27 | hsa-miR-15a* | 0.69 | 0.02 | hsa-miR-148b* | 0.71 | 0.06 |
| hsa-miR-15a* | 0.97 | 0.14 | hsa-miR-15b | 0.72 | 0.10 | hsa-miR-149 | 0.90 | 0.06 |
| hsa-miR-15b | 1.10 | 0.11 | hsa-miR-15b* | 0.76 | 0.03 | hsa-miR-149* | 1.26 | 0.07 |
| hsa-miR-15b* | 0.87 | 0.09 | hsa-miR-16 | 0.91 | 0.13 | hsa-miR-151-3p | 0.80 | 0.09 |
| hsa-miR-16 | 1.22 | 0.27 | hsa-miR-16-1* | 0.58 | 0.04 | hsa-miR-152 | 1.19 | 0.13 |
| hsa-miR-16-1* | 1.29 | 0.15 | hsa-miR-17 | 0.70 | 0.12 | hsa-miR-154* | 0.65 | 0.02 |
| hsa-miR-17 | 1.16 | 0.19 | hsa-miR-17* | 0.77 | 0.06 | hsa-miR-15a | 1.04 | 0.09 |
| hsa-miR-17* | 0.75 | 0.11 | hsa-miR-181a | 0.92 | 0.19 | hsa-miR-15a* | 0.64 | 0.06 |
| hsa-miR-181a | 1.25 | 0.08 | hsa-miR-181a-2* | 0.71 | 0.04 | hsa-miR-15b | 1.33 | 0.25 |
| hsa-miR-181a-2* | 0.76 | 0.10 | hsa-miR-182 | 1.71 | 0.45 | hsa-miR-15b* | 0.96 | 0.04 |

| **NTERA-2-R/ NTERA-2** | | | **NCCIT-R / NCCIT** | | | **2102EP-R / 2102EP** | | |
| --- | --- | --- | --- | --- | --- | --- | --- | --- |
| Detector | fold-change | SEM | Detector | fold-change | SEM | Detector | fold-change | SEM |
| hsa-miR-182 | 1.57 | 0.28 | hsa-miR-183 | 1.90 | 0.21 | hsa-miR-16 | 1.35 | 0.09 |
| hsa-miR-183 | 1.80 | 0.22 | hsa-miR-183* | 1.38 | 0.10 | hsa-miR-17 | 1.22 | 0.14 |
| hsa-miR-183* | 1.15 | 0.06 | hsa-miR-185 | 0.86 | 0.15 | hsa-miR-17* | 1.01 | 0.20 |
| hsa-miR-185 | 1.30 | 0.20 | hsa-miR-186 | 0.75 | 0.03 | hsa-miR-182 | 1.05 | 0.06 |
| hsa-miR-186 | 1.21 | 0.29 | hsa-miR-187 | 0.61 | 0.02 | hsa-miR-183 | 0.87 | 0.13 |
| hsa-miR-187 | 1.44 | 0.21 | hsa-miR-188-5p | 0.68 | 0.13 | hsa-miR-183* | 0.64 | 0.03 |
| hsa-miR-188-5p | 1.14 | 0.26 | hsa-miR-18a | 0.66 | 0.06 | hsa-miR-184 | 1.03 | 0.07 |
| hsa-miR-18a | 1.33 | 0.24 | hsa-miR-18a* | 0.79 | 0.03 | hsa-miR-185 | 1.50 | 0.08 |
| hsa-miR-18a* | 0.89 | 0.12 | hsa-miR-18b | 0.65 | 0.05 | hsa-miR-186 | 0.99 | 0.10 |
| hsa-miR-18b | 1.34 | 0.23 | hsa-miR-18b* | 0.65 | 0.13 | hsa-miR-187 | 0.74 | 0.01 |
| hsa-miR-18b* | 0.84 | 0.17 | hsa-miR-191 | 1.21 | 0.12 | hsa-miR-188-5p | 1.09 | 0.23 |
| hsa-miR-191 | 1.16 | 0.23 | hsa-miR-192 | 0.42 | 0.06 | hsa-miR-18a | 1.43 | 0.09 |
| hsa-miR-192 | 0.75 | 0.15 | hsa-miR-193a-5p | 0.68 | 0.13 | hsa-miR-18a* | 1.17 | 0.13 |
| hsa-miR-193a-5p | 1.12 | 0.11 | hsa-miR-193b | 0.68 | 0.07 | hsa-miR-18b | 1.54 | 0.19 |
| hsa-miR-193b | 2.09 | 0.49 | hsa-miR-194 | 0.48 | 0.18 | hsa-miR-191 | 1.24 | 0.03 |
| hsa-miR-194 | 0.88 | 0.21 | hsa-miR-195 | 0.63 | 0.13 | hsa-miR-192 | 1.11 | 0.16 |
| hsa-miR-195 | 1.06 | 0.28 | hsa-miR-197 | 0.84 | 0.08 | hsa-miR-193a-5p | 0.39 | 0.07 |
| hsa-miR-197 | 0.98 | 0.22 | hsa-miR-199a-3p | 1.11 | 0.13 | hsa-miR-193b | 0.63 | 0.03 |
| hsa-miR-199a-3p | 1.06 | 0.08 | hsa-miR-19a | 0.90 | 0.24 | hsa-miR-194 | 0.89 | 0.19 |
| hsa-miR-19a | 1.58 | 0.27 | hsa-miR-19b | 0.78 | 0.18 | hsa-miR-195 | 0.97 | 0.19 |
| hsa-miR-19b | 1.45 | 0.28 | hsa-miR-19b-1* | 0.75 | 0.06 | hsa-miR-197 | 1.16 | 0.31 |
| hsa-miR-19b-1* | 1.02 | 0.16 | hsa-miR-200a | 1.16 | 0.28 | hsa-miR-19a | 1.17 | 0.19 |
| hsa-miR-200a | 1.06 | 0.20 | hsa-miR-200b | 1.02 | 0.07 | hsa-miR-19b | 1.12 | 0.20 |
| hsa-miR-200b | 0.99 | 0.18 | hsa-miR-200c | 1.25 | 0.15 | hsa-miR-19b-1* | 5.73 | 3.51 |
| hsa-miR-200c | 1.15 | 0.01 | hsa-miR-204 | 0.86 | 0.01 | hsa-miR-200b | 1.04 | 0.27 |
| hsa-miR-204 | 1.11 | 0.39 | hsa-miR-205 | 0.49 | 0.14 | hsa-miR-200c | 1.19 | 0.24 |
| hsa-miR-205 | 2.87 | 0.45 | hsa-miR-20a | 0.67 | 0.10 | hsa-miR-200c* | 1.27 | 0.22 |
| hsa-miR-20a | 1.08 | 0.14 | hsa-miR-20a* | 0.79 | 0.08 | hsa-miR-204 | 1.27 | 0.11 |
| hsa-miR-20a* | 0.99 | 0.03 | hsa-miR-20b | 0.63 | 0.05 | hsa-miR-205 | 0.67 | 0.03 |
| hsa-miR-20b | 0.87 | 0.04 | hsa-miR-20b* | 0.50 | 0.04 | hsa-miR-20a | 1.60 | 0.23 |
| hsa-miR-20b* | 0.62 | 0.06 | hsa-miR-21 | 0.47 | 0.04 | hsa-miR-20a* | 1.23 | 0.05 |
| hsa-miR-21 | 1.47 | 0.25 | hsa-miR-210 | 1.00 | 0.07 | hsa-miR-20b | 0.77 | 0.12 |
| hsa-miR-210 | 1.40 | 0.30 | hsa-miR-212 | 1.05 | 0.05 | hsa-miR-20b* | 0.59 | 0.03 |
| hsa-miR-212 | 0.97 | 0.35 | hsa-miR-218 | 0.45 | 0.06 | hsa-miR-21 | 1.00 | 0.13 |
| hsa-miR-215 | 0.87 | 0.18 | hsa-miR-219-2-3p | 0.69 | 0.11 | hsa-miR-21* | 0.53 | 0.14 |
| hsa-miR-218 | 0.83 | 0.10 | hsa-miR-22 | 1.04 | 0.14 | hsa-miR-210 | 0.90 | 0.09 |
| hsa-miR-219-2-3p | 0.87 | 0.08 | hsa-miR-22* | 0.79 | 0.08 | hsa-miR-212 | 0.71 | 0.14 |
| hsa-miR-22 | 1.07 | 0.34 | hsa-miR-221 | 0.50 | 0.06 | hsa-miR-218 | 0.30 | 0.01 |
| hsa-miR-22* | 0.64 | 0.04 | hsa-miR-222 | 0.62 | 0.08 | hsa-miR-22 | 1.57 | 0.19 |
| hsa-miR-221 | 1.07 | 0.24 | hsa-miR-222* | 0.81 | 0.02 | hsa-miR-22* | 0.99 | 0.11 |
| hsa-miR-222 | 0.90 | 0.19 | hsa-miR-224 | 0.69 | 0.09 | hsa-miR-221 | 1.05 | 0.34 |
| hsa-miR-222* | 0.89 | 0.08 | hsa-miR-23b | 0.66 | 0.09 | hsa-miR-222 | 0.92 | 0.08 |
| hsa-miR-224 | 0.81 | 0.12 | hsa-miR-24 | 0.73 | 0.10 | hsa-miR-222* | 0.85 | 0.01 |
| hsa-miR-23b | 1.17 | 0.23 | hsa-miR-25 | 0.70 | 0.10 | hsa-miR-224 | 0.59 | 0.09 |
| hsa-miR-24 | 1.31 | 0.25 | hsa-miR-25* | 0.54 | 0.03 | hsa-miR-24 | 1.33 | 0.06 |
| hsa-miR-25 | 1.11 | 0.22 | hsa-miR-26a | 0.82 | 0.15 | hsa-miR-25 | 1.34 | 0.12 |
| hsa-miR-25* | 0.71 | 0.07 | hsa-miR-26a-1* | 0.80 | 0.05 | hsa-miR-25* | 1.80 | 0.33 |
| hsa-miR-26a | 0.95 | 0.15 | hsa-miR-26b | 0.94 | 0.12 | hsa-miR-26a | 0.57 | 0.10 |
| hsa-miR-26a-1* | 0.87 | 0.09 | hsa-miR-26b* | 1.17 | 0.20 | hsa-miR-26a-1* | 0.58 | 0.03 |
| hsa-miR-26b | 0.78 | 0.14 | hsa-miR-27a | 0.76 | 0.07 | hsa-miR-26b | 0.94 | 0.10 |
| hsa-miR-26b* | 0.59 | 0.14 | hsa-miR-27a* | 0.73 | 0.09 | hsa-miR-26b* | 1.16 | 0.17 |
| hsa-miR-27a | 1.43 | 0.44 | hsa-miR-27b | 0.78 | 0.11 | hsa-miR-27a | 0.97 | 0.09 |
| hsa-miR-27a* | 1.03 | 0.12 | hsa-miR-27b* | 0.60 | 0.05 | hsa-miR-27a* | 1.00 | 0.07 |
| hsa-miR-27b | 1.75 | 0.34 | hsa-miR-28-3p | 1.14 | 0.24 | hsa-miR-27b | 1.67 | 0.34 |
| hsa-miR-27b* | 0.94 | 0.10 | hsa-miR-28-5p | 1.17 | 0.19 | hsa-miR-27b* | 1.55 | 0.15 |
| hsa-miR-28-3p | 0.76 | 0.19 | hsa-miR-296-3p | 0.76 | 0.12 | hsa-miR-28-3p | 0.92 | 0.07 |
| hsa-miR-28-5p | 1.08 | 0.33 | hsa-miR-296-5p | 0.73 | 0.03 | hsa-miR-28-5p | 1.12 | 0.22 |
| hsa-miR-296-3p | 0.94 | 0.24 | hsa-miR-29a | 0.68 | 0.19 | hsa-miR-296-5p | 0.43 | 0.03 |
| hsa-miR-296-5p | 1.16 | 0.30 | hsa-miR-29c | 1.11 | 0.15 | hsa-miR-299-3p | 1.51 | 0.35 |
| hsa-miR-29a | 1.87 | 0.45 | hsa-miR-301a | 0.64 | 0.07 | hsa-miR-299-5p | 1.24 | 0.16 |

| **NTERA-2-R/ NTERA-2** | | | **NCCIT-R / NCCIT** | | | **2102EP-R / 2102EP** | | |
| --- | --- | --- | --- | --- | --- | --- | --- | --- |
| Detector | fold-change | SEM | Detector | fold-change | SEM | Detector | fold-change | SEM |
| hsa-miR-29c | 1.25 | 0.10 | hsa-miR-301b | 0.64 | 0.09 | hsa-miR-29a | 1.79 | 0.37 |
| hsa-miR-301a | 1.48 | 0.40 | hsa-miR-302a | 0.48 | 0.05 | hsa-miR-29b | 1.86 | 0.57 |
| hsa-miR-301b | 1.38 | 0.28 | hsa-miR-302a* | 0.60 | 0.09 | hsa-miR-29c | 1.53 | 0.05 |
| hsa-miR-302a | 1.12 | 0.03 | hsa-miR-302b | 0.58 | 0.08 | hsa-miR-301a | 1.12 | 0.15 |
| hsa-miR-302a* | 0.68 | 0.02 | hsa-miR-302b* | 0.48 | 0.10 | hsa-miR-301b | 1.27 | 0.19 |
| hsa-miR-302b | 1.14 | 0.15 | hsa-miR-302c | 0.52 | 0.04 | hsa-miR-302a | 1.66 | 0.41 |
| hsa-miR-302b* | 2.30 | 1.21 | hsa-miR-302c* | 0.59 | 0.03 | hsa-miR-302a* | 1.04 | 0.04 |
| hsa-miR-302c | 1.12 | 0.21 | hsa-miR-302d | 0.41 | 0.03 | hsa-miR-302b | 1.32 | 0.20 |
| hsa-miR-302c* | 0.80 | 0.06 | hsa-miR-302d | 0.56 | 0.04 | hsa-miR-302b* | 0.90 | 0.05 |
| hsa-miR-302d | 0.76 | 0.18 | hsa-miR-302d* | 0.56 | 0.12 | hsa-miR-302c | 1.21 | 0.18 |
| hsa-miR-302d | 0.72 | 0.07 | hsa-miR-30a | 1.08 | 0.16 | hsa-miR-302c* | 0.97 | 0.06 |
| hsa-miR-302d* | 0.80 | 0.14 | hsa-miR-30a* | 0.96 | 0.20 | hsa-miR-302d | 0.85 | 0.11 |
| hsa-miR-30a | 0.87 | 0.03 | hsa-miR-30b | 0.73 | 0.08 | hsa-miR-302d | 1.04 | 0.16 |
| hsa-miR-30a* | 0.67 | 0.03 | hsa-miR-30c | 0.53 | 0.02 | hsa-miR-302d* | 0.88 | 0.21 |
| hsa-miR-30b | 0.99 | 0.15 | hsa-miR-30d | 1.46 | 0.09 | hsa-miR-30a | 0.88 | 0.05 |
| hsa-miR-30c | 0.82 | 0.12 | hsa-miR-30d | 1.02 | 0.13 | hsa-miR-30a* | 1.22 | 0.09 |
| hsa-miR-30d | 1.06 | 0.22 | hsa-miR-30d* | 0.88 | 0.02 | hsa-miR-30b | 0.82 | 0.09 |
| hsa-miR-30d | 0.83 | 0.17 | hsa-miR-30e | 0.52 | 0.08 | hsa-miR-30c | 1.03 | 0.16 |
| hsa-miR-30d* | 1.38 | 0.10 | hsa-miR-30e* | 0.84 | 0.06 | hsa-miR-30d | 1.04 | 0.16 |
| hsa-miR-30e | 0.89 | 0.20 | hsa-miR-31 | 0.83 | 0.02 | hsa-miR-30d | 0.73 | 0.02 |
| hsa-miR-30e* | 0.70 | 0.04 | hsa-miR-32 | 0.67 | 0.12 | hsa-miR-30d* | 1.11 | 0.16 |
| hsa-miR-31 | 1.14 | 0.18 | hsa-miR-320 | 1.03 | 0.10 | hsa-miR-30e | 0.66 | 0.08 |
| hsa-miR-32 | 1.42 | 0.28 | hsa-miR-323-3p | 0.94 | 0.21 | hsa-miR-30e* | 0.83 | 0.05 |
| hsa-miR-320 | 1.32 | 0.20 | hsa-miR-324-3p | 0.87 | 0.08 | hsa-miR-31 | 0.67 | 0.07 |
| hsa-miR-324-3p | 0.99 | 0.11 | hsa-miR-324-5p | 0.78 | 0.07 | hsa-miR-32 | 1.13 | 0.22 |
| hsa-miR-324-5p | 1.45 | 0.25 | hsa-miR-326 | 0.70 | 0.06 | hsa-miR-320 | 1.32 | 0.08 |
| hsa-miR-326 | 1.18 | 0.21 | hsa-miR-328 | 0.77 | 0.12 | hsa-miR-323-3p | 1.05 | 0.06 |
| hsa-miR-328 | 1.02 | 0.05 | hsa-miR-330-3p | 1.18 | 0.06 | hsa-miR-324-3p | 1.59 | 0.15 |
| hsa-miR-330-3p | 0.83 | 0.11 | hsa-miR-331-3p | 0.72 | 0.04 | hsa-miR-324-5p | 1.59 | 0.34 |
| hsa-miR-331-3p | 1.10 | 0.22 | hsa-miR-331-5p | 0.64 | 0.10 | hsa-miR-328 | 0.80 | 0.07 |
| hsa-miR-331-5p | 1.09 | 0.25 | hsa-miR-335 | 0.46 | 0.06 | hsa-miR-330-3p | 1.17 | 0.04 |
| hsa-miR-335 | 1.19 | 0.18 | hsa-miR-335* | 1.02 | 0.27 | hsa-miR-331-3p | 1.10 | 0.24 |
| hsa-miR-335* | 0.86 | 0.17 | hsa-miR-339-3p | 0.94 | 0.10 | hsa-miR-331-5p | 1.71 | 0.19 |
| hsa-miR-339-3p | 1.43 | 0.20 | hsa-miR-339-5p | 1.13 | 0.16 | hsa-miR-335 | 0.74 | 0.08 |
| hsa-miR-339-5p | 1.32 | 0.18 | hsa-miR-33a* | 1.18 | 0.05 | hsa-miR-335* | 0.47 | 0.05 |
| hsa-miR-33a* | 0.82 | 0.08 | hsa-miR-340 | 1.22 | 0.25 | hsa-miR-337-5p | 1.41 | 0.09 |
| hsa-miR-340 | 1.18 | 0.21 | hsa-miR-340* | 1.02 | 0.12 | hsa-miR-339-3p | 1.08 | 0.10 |
| hsa-miR-340* | 0.51 | 0.05 | hsa-miR-342-3p | 0.77 | 0.15 | hsa-miR-339-5p | 0.72 | 0.29 |
| hsa-miR-342-3p | 1.18 | 0.24 | hsa-miR-345 | 0.94 | 0.17 | hsa-miR-33a* | 0.90 | 0.06 |
| hsa-miR-345 | 1.36 | 0.31 | hsa-miR-346 | 1.11 | 0.38 | hsa-miR-340 | 0.73 | 0.08 |
| hsa-miR-34a | 1.21 | 0.11 | hsa-miR-34a | 1.06 | 0.19 | hsa-miR-340* | 0.76 | 0.19 |
| hsa-miR-34c-5p | 0.94 | 0.30 | hsa-miR-34c-5p | 0.64 | 0.15 | hsa-miR-342-3p | 0.93 | 0.06 |
| hsa-miR-361-3p | 0.73 | 0.11 | hsa-miR-361-3p | 1.32 | 0.13 | hsa-miR-345 | 1.34 | 0.22 |
| hsa-miR-361-3p | 0.67 | 0.13 | hsa-miR-361-3p | 0.87 | 0.17 | hsa-miR-346 | 1.77 | 0.21 |
| hsa-miR-361-5p | 0.80 | 0.16 | hsa-miR-361-5p | 0.91 | 0.04 | hsa-miR-34a | 2.36 | 0.45 |
| hsa-miR-362-3p | 3.71 | 1.67 | hsa-miR-362-3p | 0.97 | 0.26 | hsa-miR-34a* | 1.60 | 0.10 |
| hsa-miR-362-5p | 1.41 | 0.12 | hsa-miR-362-5p | 0.87 | 0.10 | hsa-miR-34c-5p | 1.13 | 0.08 |
| hsa-miR-363 | 1.13 | 0.16 | hsa-miR-363 | 0.58 | 0.11 | hsa-miR-361-5p | 0.89 | 0.18 |
| hsa-miR-363* | 0.62 | 0.09 | hsa-miR-363* | 0.48 | 0.02 | hsa-miR-362-3p | 2.39 | 0.75 |
| hsa-miR-365 | 1.39 | 0.31 | hsa-miR-365 | 0.96 | 0.05 | hsa-miR-362-5p | 1.90 | 0.11 |
| hsa-miR-367 | 1.85 | 0.83 | hsa-miR-367 | 0.54 | 0.05 | hsa-miR-363 | 0.98 | 0.16 |
| hsa-miR-371-3p | 4.02 | 0.30 | hsa-miR-371-3p | 15.24 | 2.14 | hsa-miR-363* | 0.95 | 0.16 |
| hsa-miR-372 | 4.73 | 0.99 | hsa-miR-372 | 16.28 | 1.99 | hsa-miR-365 | 0.34 | 0.05 |
| hsa-miR-373 | 4.13 | 0.69 | hsa-miR-373 | 11.96 | 2.95 | hsa-miR-367 | 1.12 | 0.37 |
| hsa-miR-374a | 1.21 | 0.09 | hsa-miR-374a | 0.65 | 0.22 | hsa-miR-369-5p | 1.37 | 0.28 |
| hsa-miR-374b | 1.03 | 0.20 | hsa-miR-374b | 0.65 | 0.13 | hsa-miR-370 | 1.54 | 0.15 |
| hsa-miR-378 | 1.05 | 0.19 | hsa-miR-375 | 1.14 | 0.06 | hsa-miR-371-3p | 1.01 | 0.22 |
| hsa-miR-378* | 0.97 | 0.08 | hsa-miR-378 | 0.86 | 0.08 | hsa-miR-372 | 1.02 | 0.20 |
| hsa-miR-422a | 1.10 | 0.30 | hsa-miR-378* | 0.86 | 0.05 | hsa-miR-373 | 0.94 | 0.09 |
| hsa-miR-423-5p | 1.63 | 0.24 | hsa-miR-383 | 13.33 | 2.84 | hsa-miR-373* | 0.73 | 0.10 |

| **NTERA-2-R/ NTERA-2** | | | **NCCIT-R / NCCIT** | | | **2102EP-R / 2102EP** | | |
| --- | --- | --- | --- | --- | --- | --- | --- | --- |
| Detector | fold-change | SEM | Detector | fold-change | SEM | Detector | fold-change | SEM |
| hsa-miR-424 | 1.13 | 0.08 | hsa-miR-422a | 1.08 | 0.15 | hsa-miR-374a | 1.13 | 0.02 |
| hsa-miR-424* | 0.82 | 0.22 | hsa-miR-423-5p | 0.87 | 0.16 | hsa-miR-374b | 1.57 | 0.37 |
| hsa-miR-425 | 0.96 | 0.12 | hsa-miR-424* | 0.48 | 0.05 | hsa-miR-375 | 0.73 | 0.10 |
| hsa-miR-425* | 1.02 | 0.08 | hsa-miR-425 | 1.09 | 0.15 | hsa-miR-376a | 1.72 | 0.18 |
| hsa-miR-429 | 0.84 | 0.13 | hsa-miR-425* | 1.15 | 0.06 | hsa-miR-376c | 1.33 | 0.19 |
| hsa-miR-449a | 0.84 | 0.08 | hsa-miR-449a | 1.05 | 0.22 | hsa-miR-377* | 1.35 | 0.13 |
| hsa-miR-450b-5p | 1.24 | 0.13 | hsa-miR-450b-5p | 0.38 | 0.01 | hsa-miR-378 | 0.75 | 0.05 |
| hsa-miR-454 | 0.90 | 0.25 | hsa-miR-454 | 0.85 | 0.21 | hsa-miR-378* | 1.01 | 0.19 |
| hsa-miR-454* | 0.60 | 0.04 | hsa-miR-454* | 0.82 | 0.04 | hsa-miR-379 | 1.30 | 0.07 |
| hsa-miR-455-3p | 1.25 | 0.24 | hsa-miR-455-3p | 0.83 | 0.08 | hsa-miR-379* | 0.59 | 0.07 |
| hsa-miR-455-5p | 2.01 | 0.37 | hsa-miR-455-5p | 0.68 | 0.12 | hsa-miR-380* | 1.12 | 0.11 |
| hsa-miR-484 | 1.04 | 0.26 | hsa-miR-484 | 0.82 | 0.16 | hsa-miR-381 | 1.48 | 0.08 |
| hsa-miR-487a | 1.53 | 0.42 | hsa-miR-487a | 1.33 | 0.56 | hsa-miR-382 | 1.32 | 0.27 |
| hsa-miR-489 | 1.56 | 0.41 | hsa-miR-489 | 0.80 | 0.13 | hsa-miR-409-3p | 0.96 | 0.09 |
| hsa-miR-491-5p | 1.08 | 0.05 | hsa-miR-491-5p | 1.02 | 0.14 | hsa-miR-409-3p | 0.80 | 0.09 |
| hsa-miR-494 | 1.79 | 0.79 | hsa-miR-494 | 1.28 | 0.49 | hsa-miR-409-5p | 1.01 | 0.19 |
| hsa-miR-500 | 1.45 | 0.18 | hsa-miR-497 | 0.84 | 0.07 | hsa-miR-410 | 0.90 | 0.02 |
| hsa-miR-500* | 0.76 | 0.09 | hsa-miR-500 | 0.85 | 0.04 | hsa-miR-411 | 1.16 | 0.13 |
| hsa-miR-501-3p | 1.27 | 0.12 | hsa-miR-500* | 1.46 | 0.08 | hsa-miR-411* | 0.71 | 0.11 |
| hsa-miR-501-5p | 1.60 | 0.11 | hsa-miR-501-5p | 1.10 | 0.26 | hsa-miR-422a | 1.25 | 0.03 |
| hsa-miR-502-3p | 1.51 | 0.25 | hsa-miR-502-3p | 0.90 | 0.12 | hsa-miR-423-5p | 1.34 | 0.10 |
| hsa-miR-502-5p | 1.14 | 0.17 | hsa-miR-502-5p | 1.08 | 0.25 | hsa-miR-424 | 1.32 | 0.14 |
| hsa-miR-503 | 1.38 | 0.03 | hsa-miR-503 | 0.73 | 0.08 | hsa-miR-424* | 0.65 | 0.14 |
| hsa-miR-504 | 0.76 | 0.22 | hsa-miR-504 | 0.56 | 0.03 | hsa-miR-425 | 1.18 | 0.10 |
| hsa-miR-505 | 0.89 | 0.11 | hsa-miR-505 | 0.69 | 0.08 | hsa-miR-425* | 1.08 | 0.03 |
| hsa-miR-505* | 0.59 | 0.09 | hsa-miR-505* | 0.86 | 0.12 | hsa-miR-431 | 1.34 | 0.12 |
| hsa-miR-512-3p | 5.06 | 0.93 | hsa-miR-512-3p | 7.79 | 1.21 | hsa-miR-432 | 1.47 | 0.22 |
| hsa-miR-512-5p | 4.62 | 1.31 | hsa-miR-512-5p | 6.26 | 0.83 | hsa-miR-432 | 1.55 | 0.14 |
| hsa-miR-515-3p | 6.37 | 0.22 | hsa-miR-515-3p | 4.79 | 0.53 | hsa-miR-432* | 1.37 | 0.08 |
| hsa-miR-515-5p | 7.79 | 0.96 | hsa-miR-515-5p | 5.64 | 0.27 | hsa-miR-433 | 1.80 | 0.24 |
| hsa-miR-516a-3p | 1.14 | 0.14 | hsa-miR-516b | 3.14 | 0.52 | hsa-miR-452 | 0.73 | 0.03 |
| hsa-miR-516b | 4.52 | 0.39 | hsa-miR-517a | 5.99 | 0.60 | hsa-miR-453 | 1.29 | 0.10 |
| hsa-miR-517a | 5.26 | 1.18 | hsa-miR-517b | 6.44 | 1.52 | hsa-miR-454 | 1.65 | 0.24 |
| hsa-miR-517b | 7.18 | 1.25 | hsa-miR-517c | 6.69 | 0.48 | hsa-miR-454* | 0.99 | 0.08 |
| hsa-miR-517c | 6.14 | 0.49 | hsa-miR-518a-3p | 7.19 | 0.34 | hsa-miR-455-3p | 1.52 | 0.21 |
| hsa-miR-518a-3p | 6.87 | 1.13 | hsa-miR-518b | 8.14 | 1.22 | hsa-miR-455-5p | 1.31 | 0.18 |
| hsa-miR-518b | 7.27 | 0.39 | hsa-miR-518c | 1.70 | 0.22 | hsa-miR-484 | 0.43 | 0.03 |
| hsa-miR-518c | 2.88 | 0.43 | hsa-miR-518d-5p | 3.28 | 0.39 | hsa-miR-485-3p | 1.31 | 0.22 |
| hsa-miR-518d-5p | 4.46 | 0.54 | hsa-miR-518e | 8.14 | 1.49 | hsa-miR-485-5p | 1.55 | 0.19 |
| hsa-miR-518e | 6.68 | 0.39 | hsa-miR-518f | 6.29 | 0.87 | hsa-miR-486-5p | 1.57 | 0.02 |
| hsa-miR-518e* | 1.92 | 0.33 | hsa-miR-519a | 6.02 | 0.95 | hsa-miR-487a | 0.98 | 0.07 |
| hsa-miR-518f | 5.17 | 0.67 | hsa-miR-519b-3p | 7.83 | 1.01 | hsa-miR-487b | 1.19 | 0.04 |
| hsa-miR-519a | 6.78 | 1.00 | hsa-miR-519b-3p | 8.60 | 1.31 | hsa-miR-489 | 0.10 | 0.01 |
| hsa-miR-519b-3p | 4.44 | 0.41 | hsa-miR-519c-3p | 3.24 | 0.27 | hsa-miR-491-5p | 0.97 | 0.06 |
| hsa-miR-519b-3p | 6.85 | 1.32 | hsa-miR-519d | 7.05 | 2.65 | hsa-miR-493 | 1.59 | 0.12 |
| hsa-miR-519c-3p | 4.25 | 0.34 | hsa-miR-520a-5p | 1.60 | 0.17 | hsa-miR-494 | 1.24 | 0.07 |
| hsa-miR-519d | 5.25 | 0.74 | hsa-miR-520b | 5.47 | 0.81 | hsa-miR-495 | 0.70 | 0.13 |
| hsa-miR-520a-5p | 1.96 | 0.34 | hsa-miR-520c-3p | 5.64 | 0.53 | hsa-miR-500 | 1.67 | 0.22 |
| hsa-miR-520b | 6.98 | 2.31 | hsa-miR-520f | 2.38 | 0.31 | hsa-miR-500* | 0.85 | 0.05 |
| hsa-miR-520c-3p | 4.64 | 0.65 | hsa-miR-520g | 7.53 | 1.49 | hsa-miR-501-5p | 1.66 | 0.12 |
| hsa-miR-520f | 3.73 | 0.25 | hsa-miR-520h | 7.14 | 0.72 | hsa-miR-502-3p | 1.87 | 0.33 |
| hsa-miR-520g | 5.84 | 0.62 | hsa-miR-521 | 1.59 | 0.38 | hsa-miR-502-5p | 1.87 | 0.09 |
| hsa-miR-520h | 3.88 | 0.36 | hsa-miR-522 | 6.45 | 0.44 | hsa-miR-503 | 1.24 | 0.12 |
| hsa-miR-521 | 1.90 | 0.44 | hsa-miR-523 | 2.99 | 0.50 | hsa-miR-504 | 0.56 | 0.07 |
| hsa-miR-522 | 4.29 | 0.72 | hsa-miR-525-3p | 7.97 | 0.92 | hsa-miR-505 | 1.52 | 0.45 |
| hsa-miR-523 | 3.49 | 0.14 | hsa-miR-526b* | 8.31 | 2.27 | hsa-miR-505* | 1.01 | 0.10 |
| hsa-miR-524-3p | 2.40 | 0.06 | hsa-miR-532-3p | 0.88 | 0.09 | hsa-miR-508-3p | 1.56 | 0.30 |
| hsa-miR-525-3p | 6.78 | 1.32 | hsa-miR-532-5p | 0.77 | 0.12 | hsa-miR-509-3p | 0.68 | 0.05 |
| hsa-miR-525-5p | 1.97 | 0.33 | hsa-miR-550 | 0.89 | 0.19 | hsa-miR-509-5p | 1.93 | 0.38 |
| hsa-miR-526b | 2.23 | 0.43 | hsa-miR-550* | 1.00 | 0.14 | hsa-miR-512-3p | 2.07 | 0.21 |

| **NTERA-2-R/ NTERA-2** | | | **NCCIT-R / NCCIT** | | | **2102EP-R / 2102EP** | | |
| --- | --- | --- | --- | --- | --- | --- | --- | --- |
| Detector | fold-change | SEM | Detector | fold-change | SEM | Detector | fold-change | SEM |
| hsa-miR-526b* | 4.31 | 0.48 | hsa-miR-565 | 0.76 | 0.03 | hsa-miR-512-5p | 1.51 | 0.16 |
| hsa-miR-532-3p | 1.31 | 0.16 | hsa-miR-574-3p | 0.61 | 0.11 | hsa-miR-515-3p | 1.41 | 0.23 |
| hsa-miR-532-5p | 1.78 | 0.19 | hsa-miR-576-3p | 0.47 | 0.06 | hsa-miR-515-5p | 1.57 | 0.16 |
| hsa-miR-548b-5p | 0.96 | 0.08 | hsa-miR-579 | 0.60 | 0.10 | hsa-miR-516a-3p | 1.03 | 0.13 |
| hsa-miR-550 | 0.80 | 0.08 | hsa-miR-582-5p | 0.20 | 0.04 | hsa-miR-516a-5p | 1.09 | 0.10 |
| hsa-miR-550* | 0.97 | 0.11 | hsa-miR-589* | 1.56 | 0.12 | hsa-miR-516b | 1.10 | 0.15 |
| hsa-miR-565 | 0.84 | 0.08 | hsa-miR-590-5p | 1.17 | 0.14 | hsa-miR-517* | 0.98 | 0.08 |
| hsa-miR-570 | 1.45 | 0.17 | hsa-miR-592 | 1.50 | 0.23 | hsa-miR-517a | 1.61 | 0.13 |
| hsa-miR-574-3p | 1.03 | 0.33 | hsa-miR-597 | 0.86 | 0.18 | hsa-miR-517b | 1.86 | 0.67 |
| hsa-miR-576-3p | 0.96 | 0.18 | hsa-miR-598 | 0.92 | 0.06 | hsa-miR-517c | 1.27 | 0.07 |
| hsa-miR-579 | 1.26 | 0.23 | hsa-miR-625 | 0.91 | 0.00 | hsa-miR-518a-3p | 1.01 | 0.06 |
| hsa-miR-582-5p | 1.26 | 0.23 | hsa-miR-625* | 1.22 | 0.48 | hsa-miR-518b | 1.70 | 0.08 |
| hsa-miR-589* | 1.15 | 0.17 | hsa-miR-628-5p | 1.16 | 0.17 | hsa-miR-518c | 1.58 | 0.42 |
| hsa-miR-590-5p | 1.80 | 0.65 | hsa-miR-629* | 1.14 | 0.14 | hsa-miR-518c* | 0.95 | 0.20 |
| hsa-miR-597 | 1.28 | 0.04 | hsa-miR-636 | 0.89 | 0.13 | hsa-miR-518d-3p | 1.39 | 0.13 |
| hsa-miR-598 | 1.10 | 0.09 | hsa-miR-638 | 0.97 | 0.42 | hsa-miR-518d-5p | 1.29 | 0.21 |
| hsa-miR-605 | 0.56 | 0.14 | hsa-miR-642 | 1.44 | 0.35 | hsa-miR-518e | 0.98 | 0.11 |
| hsa-miR-605 | 0.58 | 0.09 | hsa-miR-650 | 1.67 | 0.06 | hsa-miR-518e* | 1.10 | 0.05 |
| hsa-miR-616* | 0.58 | 0.01 | hsa-miR-650 | 1.26 | 0.38 | hsa-miR-518f | 1.17 | 0.12 |
| hsa-miR-625 | 0.90 | 0.05 | hsa-miR-652 | 0.75 | 0.01 | hsa-miR-518f* | 0.56 | 0.25 |
| hsa-miR-625* | 0.77 | 0.23 | hsa-miR-660 | 0.75 | 0.17 | hsa-miR-519a | 1.38 | 0.31 |
| hsa-miR-628-5p | 1.12 | 0.20 | hsa-miR-661 | 1.03 | 0.19 | hsa-miR-519b-3p | 1.20 | 0.12 |
| hsa-miR-629* | 1.13 | 0.14 | hsa-miR-671-3p | 0.71 | 0.09 | hsa-miR-519b-3p | 1.14 | 0.13 |
| hsa-miR-636 | 1.15 | 0.23 | hsa-miR-675 | 1.03 | 0.11 | hsa-miR-519c-3p | 1.69 | 0.63 |
| hsa-miR-642 | 0.94 | 0.05 | hsa-miR-675 | 1.21 | 0.12 | hsa-miR-519d | 1.59 | 0.29 |
| hsa-miR-650 | 1.00 | 0.21 | hsa-miR-7 | 0.75 | 0.14 | hsa-miR-519e | 1.43 | 0.18 |
| hsa-miR-652 | 1.02 | 0.12 | hsa-miR-7 | 0.91 | 0.25 | hsa-miR-519e* | 0.76 | 0.11 |
| hsa-miR-660 | 1.90 | 0.09 | hsa-miR-708 | 0.40 | 0.05 | hsa-miR-520a-3p | 1.22 | 0.05 |
| hsa-miR-671-3p | 0.95 | 0.14 | hsa-miR-7-1* | 0.95 | 0.05 | hsa-miR-520a-5p | 1.69 | 0.19 |
| hsa-miR-675 | 1.26 | 0.09 | hsa-miR-744 | 0.78 | 0.03 | hsa-miR-520b | 1.12 | 0.35 |
| hsa-miR-675 | 1.33 | 0.10 | hsa-miR-744* | 0.60 | 0.04 | hsa-miR-520c-3p | 1.10 | 0.14 |
| hsa-miR-7 | 0.67 | 0.02 | hsa-miR-760 | 1.06 | 0.27 | hsa-miR-520e | 1.07 | 0.06 |
| hsa-miR-7 | 0.74 | 0.06 | hsa-miR-766 | 1.29 | 0.06 | hsa-miR-520f | 1.56 | 0.23 |
| hsa-miR-708 | 0.45 | 0.11 | hsa-miR-766 | 1.11 | 0.13 | hsa-miR-520g | 1.79 | 0.33 |
| hsa-miR-7-1* | 0.72 | 0.04 | hsa-miR-768-3p | 0.90 | 0.04 | hsa-miR-520h | 0.92 | 0.08 |
| hsa-miR-744 | 0.91 | 0.11 | hsa-miR-768-3p | 0.87 | 0.04 | hsa-miR-521 | 1.34 | 0.37 |
| hsa-miR-744* | 0.73 | 0.14 | hsa-miR-769-5p | 1.10 | 0.08 | hsa-miR-522 | 1.18 | 0.25 |
| hsa-miR-760 | 1.12 | 0.14 | hsa-miR-769-5p | 0.93 | 0.09 | hsa-miR-523 | 2.00 | 0.30 |
| hsa-miR-766 | 0.84 | 0.12 | hsa-miR-801 | 1.10 | 0.30 | hsa-miR-524-3p | 1.14 | 0.07 |
| hsa-miR-766 | 0.96 | 0.11 | hsa-miR-801 | 1.15 | 0.34 | hsa-miR-524-3p | 0.78 | 0.08 |
| hsa-miR-768-3p | 0.91 | 0.11 | hsa-miR-874 | 0.55 | 0.14 | hsa-miR-525-3p | 1.43 | 0.13 |
| hsa-miR-768-3p | 0.92 | 0.08 | hsa-miR-877 | 1.03 | 0.29 | hsa-miR-525-5p | 1.23 | 0.20 |
| hsa-miR-769-5p | 0.93 | 0.11 | hsa-miR-886-3p | 0.65 | 0.10 | hsa-miR-526b | 1.24 | 0.11 |
| hsa-miR-769-5p | 0.92 | 0.04 | hsa-miR-886-5p | 1.02 | 0.08 | hsa-miR-526b* | 1.16 | 0.11 |
| hsa-miR-801 | 3.37 | 1.30 | hsa-miR-9 | 1.57 | 0.01 | hsa-miR-532-3p | 1.62 | 0.20 |
| hsa-miR-801 | 3.00 | 1.04 | hsa-miR-9* | 1.31 | 0.08 | hsa-miR-532-5p | 1.42 | 0.21 |
| hsa-miR-874 | 0.65 | 0.16 | hsa-miR-923 | 1.37 | 0.21 | hsa-miR-539 | 1.32 | 0.11 |
| hsa-miR-877 | 1.32 | 0.14 | hsa-miR-923 | 1.58 | 0.23 | hsa-miR-543 | 0.88 | 0.14 |
| hsa-miR-886-3p | 1.27 | 0.23 | hsa-miR-92a | 0.59 | 0.02 | hsa-miR-550 | 0.62 | 0.07 |
| hsa-miR-886-5p | 0.98 | 0.07 | hsa-miR-92a-1* | 0.72 | 0.05 | hsa-miR-550* | 0.59 | 0.08 |
| hsa-miR-9 | 1.66 | 0.28 | hsa-miR-92b* | 0.82 | 0.17 | hsa-miR-565 | 0.85 | 0.11 |
| hsa-miR-9* | 1.16 | 0.06 | hsa-miR-93 | 0.71 | 0.05 | hsa-miR-566 | 1.07 | 0.10 |
| hsa-miR-923 | 0.96 | 0.08 | hsa-miR-93* | 0.94 | 0.04 | hsa-miR-571 | 1.12 | 0.12 |
| hsa-miR-923 | 1.44 | 0.35 | hsa-miR-935 | 2.52 | 0.12 | hsa-miR-572 | 1.17 | 0.11 |
| hsa-miR-92a | 0.99 | 0.17 | hsa-miR-935 | 2.49 | 0.19 | hsa-miR-574-3p | 1.11 | 0.20 |
| hsa-miR-92a-1* | 0.80 | 0.12 | hsa-miR-939 | 0.82 | 0.18 | hsa-miR-576-3p | 0.96 | 0.01 |
| hsa-miR-92b* | 0.83 | 0.12 | hsa-miR-941 | 0.73 | 0.07 | hsa-miR-579 | 0.88 | 0.16 |
| hsa-miR-93 | 1.15 | 0.19 | hsa-miR-942 | 1.07 | 0.13 | hsa-miR-589* | 0.96 | 0.01 |
| hsa-miR-93* | 0.85 | 0.08 | hsa-miR-95 | 1.53 | 0.33 | hsa-miR-590-5p | 1.43 | 0.33 |
| hsa-miR-935 | 1.00 | 0.09 | hsa-miR-96 | 1.11 | 0.29 | hsa-miR-598 | 1.37 | 0.34 |

| **NTERA-2-R/ NTERA-2** | | | **NCCIT-R / NCCIT** | | | **2102EP-R / 2102EP** | | |
| --- | --- | --- | --- | --- | --- | --- | --- | --- |
| Detector | fold-change | SEM | Detector | fold-change | SEM | Detector | fold-change | SEM |
| hsa-miR-935 | 1.17 | 0.04 | hsa-miR-99a | 0.12 | 0.01 | hsa-miR-605 | 0.70 | 0.24 |
| hsa-miR-939 | 1.01 | 0.14 | hsa-miR-99b | 1.07 | 0.11 | hsa-miR-605 | 0.51 | 0.09 |
| hsa-miR-941 | 0.95 | 0.08 | hsa-miR-99b* | 1.58 | 0.28 | hsa-miR-625 | 1.85 | 0.09 |
| hsa-miR-942 | 0.62 | 0.05 |  |  |  | hsa-miR-625* | 0.92 | 0.16 |
| hsa-miR-95 | 0.87 | 0.19 |  |  |  | hsa-miR-628-5p | 1.09 | 0.09 |
| hsa-miR-96 | 1.66 | 0.08 |  |  |  | hsa-miR-629* | 0.76 | 0.15 |
| hsa-miR-99a | 1.51 | 0.44 |  |  |  | hsa-miR-636 | 1.36 | 0.24 |
| hsa-miR-99b | 1.14 | 0.32 |  |  |  | hsa-miR-650 | 1.18 | 0.23 |
| hsa-miR-99b* | 0.84 | 0.21 |  |  |  | hsa-miR-650 | 1.11 | 0.06 |
|  |  |  |  |  |  | hsa-miR-652 | 0.86 | 0.20 |
|  |  |  |  |  |  | hsa-miR-654-3p | 1.56 | 0.32 |
|  |  |  |  |  |  | hsa-miR-654-5p | 1.44 | 0.28 |
|  |  |  |  |  |  | hsa-miR-655 | 1.15 | 0.08 |
|  |  |  |  |  |  | hsa-miR-656 | 1.10 | 0.22 |
|  |  |  |  |  |  | hsa-miR-656 | 0.96 | 0.20 |
|  |  |  |  |  |  | hsa-miR-660 | 1.67 | 0.36 |
|  |  |  |  |  |  | hsa-miR-661 | 1.57 | 0.26 |
|  |  |  |  |  |  | hsa-miR-668 | 0.51 | 0.07 |
|  |  |  |  |  |  | hsa-miR-671-3p | 1.27 | 0.18 |
|  |  |  |  |  |  | hsa-miR-675 | 1.24 | 0.29 |
|  |  |  |  |  |  | hsa-miR-675 | 1.32 | 0.30 |
|  |  |  |  |  |  | hsa-miR-7 | 0.91 | 0.09 |
|  |  |  |  |  |  | hsa-miR-7 | 0.97 | 0.07 |
|  |  |  |  |  |  | hsa-miR-708 | 0.62 | 0.08 |
|  |  |  |  |  |  | hsa-miR-7-1* | 0.62 | 0.05 |
|  |  |  |  |  |  | hsa-miR-744 | 0.94 | 0.08 |
|  |  |  |  |  |  | hsa-miR-744* | 0.85 | 0.10 |
|  |  |  |  |  |  | hsa-miR-758 | 1.28 | 0.22 |
|  |  |  |  |  |  | hsa-miR-760 | 1.28 | 0.19 |
|  |  |  |  |  |  | hsa-miR-766 | 0.59 | 0.07 |
|  |  |  |  |  |  | hsa-miR-766 | 0.50 | 0.01 |
|  |  |  |  |  |  | hsa-miR-768-3p | 0.86 | 0.03 |
|  |  |  |  |  |  | hsa-miR-768-3p | 0.77 | 0.06 |
|  |  |  |  |  |  | hsa-miR-769-5p | 0.69 | 0.01 |
|  |  |  |  |  |  | hsa-miR-769-5p | 0.69 | 0.02 |
|  |  |  |  |  |  | hsa-miR-770-5p | 0.84 | 0.14 |
|  |  |  |  |  |  | hsa-miR-801 | 1.33 | 0.35 |
|  |  |  |  |  |  | hsa-miR-801 | 1.21 | 0.30 |
|  |  |  |  |  |  | hsa-miR-877 | 1.58 | 0.30 |
|  |  |  |  |  |  | hsa-miR-889 | 1.62 | 0.16 |
|  |  |  |  |  |  | hsa-miR-9 | 1.24 | 0.17 |
|  |  |  |  |  |  | hsa-miR-9* | 1.02 | 0.14 |
|  |  |  |  |  |  | hsa-miR-923 | 2.42 | 0.63 |
|  |  |  |  |  |  | hsa-miR-923 | 2.19 | 0.72 |
|  |  |  |  |  |  | hsa-miR-92a | 1.20 | 0.22 |
|  |  |  |  |  |  | hsa-miR-92a-1* | 1.31 | 0.05 |
|  |  |  |  |  |  | hsa-miR-92b* | 1.21 | 0.12 |
|  |  |  |  |  |  | hsa-miR-93 | 0.84 | 0.15 |
|  |  |  |  |  |  | hsa-miR-93* | 0.77 | 0.11 |
|  |  |  |  |  |  | hsa-miR-939 | 0.90 | 0.12 |
|  |  |  |  |  |  | hsa-miR-941 | 0.64 | 0.09 |
|  |  |  |  |  |  | hsa-miR-942 | 0.99 | 0.15 |
|  |  |  |  |  |  | hsa-miR-95 | 1.38 | 0.22 |
|  |  |  |  |  |  | hsa-miR-96 | 0.67 | 0.12 |
|  |  |  |  |  |  | hsa-miR-99a | 0.67 | 0.12 |
|  |  |  |  |  |  | hsa-miR-99b | 0.96 | 0.16 |
|  |  |  |  |  |  | hsa-miR-99b* | 0.86 | 0.08 |
